# Fast Self-Healing at Room Temperature in Diels–Alder Elastomers

**DOI:** 10.3390/polym15173527

**Published:** 2023-08-24

**Authors:** Ali Safaei, Joost Brancart, Zhanwei Wang, Sogol Yazdani, Bram Vanderborght, Guy Van Assche, Seppe Terryn

**Affiliations:** 1 Physical Chemistry and Polymer Science, Department of Materials and Chemistry, Vrije Universiteit Brussel, Pleinlaan 2, B-1050 Brussels, Belgium; ali.safaei@vub.be (A.S.); joost.brancart@vub.be (J.B.); sogol.yazdani@vub.be (S.Y.); guy.van.assche@vub.be (G.V.A.); 2 Brubotics, Vrije Universiteit Brussel and Imec, Pleinlaan 2, B-1050 Brussels, Belgium; zhanwei.wang@vub.be (Z.W.); bram.vanderborght@vub.be (B.V.)

**Keywords:** self-healing polymers, Diels–Alder, reversible polymer networks, autonomous self-healing, room temperature self-healing

## Abstract

Despite being primarily categorized as non-autonomous self-healing polymers, we demonstrate the ability of Diels–Alder polymers to heal macroscopic damages at room temperature, resulting in complete restoration of their mechanical properties within a few hours. Moreover, we observe immediate partial recovery, occurring mere minutes after reuniting the fractured surfaces. This fast room-temperature healing is accomplished by employing an off-stoichiometric maleimide-to-furan ratio in the polymer network. Through an extensive investigation of seven Diels–Alder polymers, the influence of crosslink density on self-healing, thermal, and (thermo-)mechanical performance was thoroughly examined. Crosslink density variations were achieved by adjusting the molecular weight of the monomers or utilizing the off-stoichiometric maleimide-to-furan ratio. Quasistatic tensile testing, dynamic mechanical analysis, dynamic rheometry, differential scanning calorimetry, and thermogravimetric analysis were employed to evaluate the individual effects of these parameters on material performance. While lowering the crosslink density in the polymer network via decreasing the off-stoichiometric ratio demonstrated the greatest acceleration of healing, it also led to a slight decrease in (dynamic) mechanical performance. On the other hand, reducing crosslink density using longer monomers resulted in faster healing, albeit to a lesser extent, while maintaining the (dynamic) mechanical performance.

## 1. Introduction

In light of the prevailing sustainability goals, extending the lifespan of products is of paramount importance. Consequently, there is a growing emphasis on the development of self-healing polymers, which possess the capability to fully recover from substantial damage. In the first classification, self-healing polymers are classified as extrinsic [1] or intrinsic [2]. In extrinsic self-healing polymers, the healing process relies on the release and solidification of a healing agent that is extrinsically encapsulated within the polymer network (e.g., microcapsules, nanocapsules, or vascular network). However, this approach has drawbacks: the healing capacity diminishes with repeated damage–healing cycles, and the material properties may not be identical to the original in the healed area. In contrast, intrinsic self-healing polymers, such as those utilized in this study, employ inherently reversible chemistries and can theoretically heal an infinite number of times. In a secondary classification, self-healing polymers can be categorized as either autonomous or non-autonomous. The majority of extrinsic self-healing polymers exhibit autonomous healing capabilities, whereas intrinsic self-healing polymers can be further classified into autonomous or non-autonomous categories. Autonomous self-healing polymers can heal under ambient conditions without requiring any external stimulus. On the other hand, non-autonomous self-healing polymers necessitate an external stimulus, typically heat, to activate the healing process. However, it should be noted that the autonomous healing ability is not solely dependent on the healing mechanism but also on the composition of the polymeric network in which it is incorporated. Our paper demonstrates that these factors should not be overlooked.

Within the domain of intrinsic self-healing polymers, a fundamental trade-off exists between mechanical strength/stability and the time/temperature required for healing [3,4]. Generally, polymers with higher bond strengths [5], such as those formed through dissociative covalent interactions like Diels–Alder bonds or associative covalent interactions [6] like disulfide bonds [7] and transesterifications [8], exhibit superior mechanical strength and stability due to the increased energy required to break these bonds. However, this also implies that a greater amount of energy must be supplied by the healing stimulus, resulting in slower healing mechanisms or even infeasibility at ambient temperatures. Conversely, the total bond energy of physical crosslinks [9], like hydrogen bond crosslinks [10] and coordination complexes [11,12,13], is significantly lower, necessitating less energy for their disruption. As a result, these physico-chemical networks can heal damage at lower or ambient temperatures. Nevertheless, the lower total bond energy has adverse effects on the mechanical properties of the polymers. Physically crosslinked self-healing polymers tend to have reduced mechanical strength, and in certain cases, they may exhibit creep behavior and non-negligible self-adhesion. These factors can lead to permanent plastic deformations and suboptimal strain recovery.

It is crucial to acknowledge that the healing characteristics of self-healing materials are not solely determined by the type of reversible bond they possess. The composition of their network also plays a significant role, an aspect that is often overlooked and underestimated in the existing literature. Therefore, the primary focus of this paper is to explore the impact of altering the network composition on enhancing the healing capabilities of these materials. We employ the extensively utilized reversible Diels–Alder reaction to synthesize our self-healing polymers. Compared to other reversible bonds, the Diels–Alder bond possesses a moderate strength, which leads to an outstanding balance between mechanical strength, stability, and healing time on the order of hours at mild temperatures [14]. Therefore, they have been used to introduce healing capacities in multiple applications, including soft robotics [15,16], flexible electronics [17], and coatings [18]. While the self-healing capability of furan and maleimide-based Diels–Alder polymers have been extensively documented, the majority of self-healing procedures described involve non-autonomous healing at temperatures ranging from 80 to 130 °C. However, in most applications, the need for external heat stimulus to activate the healing process complicates the system. Hence, the demand for autonomous healing under application conditions, predominantly at ambient conditions, is significant. In an initial proof-of-concept study, the authors have successfully demonstrated that by modifying the network composition to an off-stoichiometric maleimide-to-furan ratio, Diels–Alder polymers can autonomously undergo self-healing even under ambient conditions [19]. Building upon this discovery, this research delves into an in-depth investigation of methods to expedite the self-healing process. Alternatively, there have been reports on using the Diels–Alder chemistry in autonomous extrinsic self-healing polymers [20]. 

**Figure 1:**
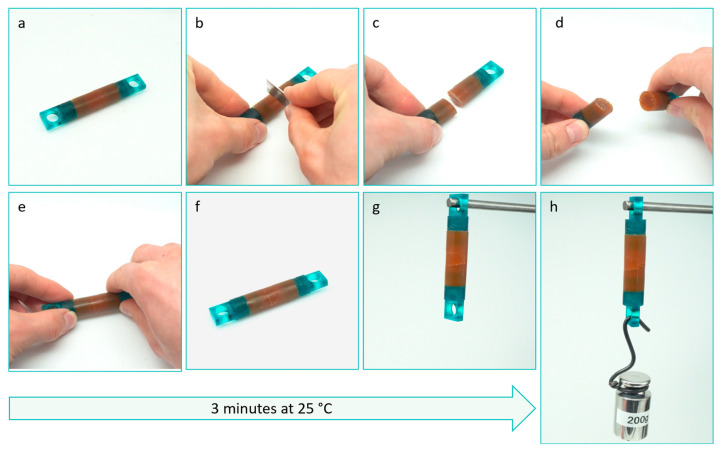
Qualitative illustration of the fast room temperature self-healing in a DPBM-FT5000-r0.5 sample: (**a**) A cylindrical sample with a diameter of 10 mm and length of 60 mm (**b**–**d**) is cut in half using a razor blade. (**e**) The fracture surfaces are manually reconnected within seconds. (**f**) The reconnected sample is left to heal at room temperature (25 °C) for only 3 min. (**g**) Subsequently, it is positioned vertically. (**h**) After 3 min of self-healing at ambient conditions, the sample can hold a weight of 200 g. The video can be found at the following link: https://www.youtube.com/watch?v=a-bCetuPKjs (accessible since 20 July 2023).

The primary enabling factor for our study is the significant advantage of the tunability of Diels–Alder polymers. Previous studies have demonstrated a remarkable range of mechanical properties, spanning from highly flexible elastomers with Young’s modulus in the 100 kPa range to rigid thermosets with moduli reaching a few GPa [21]. Achieving this versatility involves manipulating various network design parameters. These parameters include adjusting the molecular weight of the monomer units [21], controlling the functionality of the monomers [22] (e.g., the number of reactive maleimides or furan per monomer unit), and tuning the stoichiometric ratio between furan and maleimide [23,24,25]. Furthermore, blending monomers of the same type but with different molecular weights can create polymer network blends. Through blending, the resulting self-healing Diels–Alder polymers may exhibit partial phase separation within the network, leading to enhanced toughness and stiffness. In this paper, we demonstrate that these network design parameters, particularly the molecular weight of the monomer and the extent of off-stoichiometry, can also accelerate the healing process under ambient conditions. This acceleration leads to autonomous healing within minutes at room temperature (Figure 1). 

Diels–Alder polymers possess an additional advantage in terms of their remarkable manufacturability. Unlike classical elastomers and thermosets, which are characterized by irreversible crosslinks in their network structure, Diels–Alder polymers can undergo thermal reprocessing. This unique feature not only presents significant potential for material recycling but also enables a wide array of processing techniques to be employed [14]. These techniques encompass formative manufacturing methods such as casting [26], injection molding [27], and compression molding [28], as well as additive manufacturing approaches including fused filament fabrication [29,30], direct ink writing [31], and selective laser sintering [32]. Moreover, assembly and binding techniques can also be utilized with these polymers [14,15]. 

To optimize the healing process, it is crucial to understand the specific factors that contribute to its enhancement when modifying the network composition: (i) Firstly, it is essential to have a significant quantity of reactive furan and maleimide groups present on the surfaces of fractures to facilitate the formation of Diels–Alder bonds through reaction. When damage occurs, the reversible Diels–Alder bonds break along the fracture line since, although relatively strong, they are the weakest covalent bond within the network. By increasing the concentration of Diels–Alder bonds in the network (e.g., the crosslink density), a greater number of bonds will break during damage, resulting in increased availability of reactive furan and maleimide groups at the fracture surfaces. (ii) Secondly, network mobility plays a vital role in the healing process. On a microscopic level, increased mobility leads to improved contact between the fracture surfaces. Furthermore, at a molecular level, this increased mobility facilitates the encounter between reactive furan and maleimide groups, making it easier for them to find suitable binding partners and form Diels–Alder bonds. The network mobility is increased upon decreasing the Diels–Alder crosslink density. The inverse relationship between these two contributions is evident, highlighting the necessity for a comprehensive study to determine the relative importance of each factor in the context of autonomous self-healing.

Two methods can be employed to modify the Diels–Alder crosslink density in Diels–Alder polymers. The first method involves adjusting the molecular weight of the monomers within the network, while the second method involves varying the off-stoichiometric ratio of maleimide to furan in the network. To investigate the impact of these network design parameters on thermal characteristics and (thermo-)mechanical performance, a series of tests were conducted on two sets of materials comprising four and three Diels–Alder polymers. The tests included quasistatic tensile testing, dynamic mechanical analysis (DMA), dynamic rheometry, differential scanning calorimetry (DSC), and thermogravimetric analysis (TGA). Furthermore, the influence of these parameters on the self-healing capabilities was analyzed. The series of materials were subjected to quasistatic tensile testing until fracture, both before and after healing at room temperature (25 °C) for various durations ranging from 5 min to 24 h. This allowed for the observation of the evolution of Young’s modulus, stress at fracture, strain at fracture, and toughness as a function of healing time, as well as the resulting healing efficiencies. Notably, these tests involved healing significant damages, which involved cutting the tensile samples in half using a scalpel blade.

## 2. Materials and Methods 

### 2.1. Materials 

#### 2.1.1. Reagents

**Figure 2:**
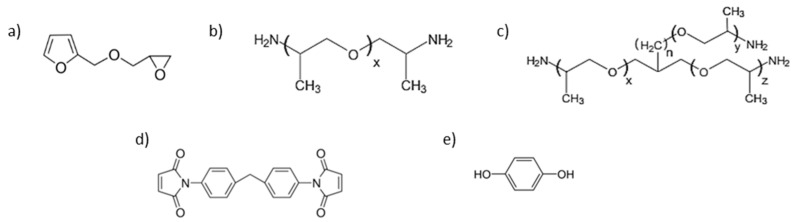
Monomers of the Diels–Alder polymer networks: (**a**) The furfuryl glycidyl ether (FGE). (**b**) Chemical structure of the diamine Jeffamines D4000 (x = 49.0) and D2000. (**c**) Chemical structure of the triamine Jeffamines T5000 (x + y + z = 96.8, n = 0) and T3000 (x + y + z = 54.2, n = 0). (**d**) The 1,1′-(methylenedi-4,1-phenylene) bismaleimide (DPBM). (**e**) Hydroquinone (1,4-benzenediol).

Furfuryl glycidyl ether (FGE) was purchased from BLD Pharmatech GmbH (Kaiserslautern, Germany), with a molecular weight of 154.16 g mol^−1^ and 97% purity. Jeffamine D4000, D2000, T5000, and T3000, with an average molecular weight of 4546, 1793, 5709, and 3180 g mol^−1^, respectively, were kindly provided by Huntsman (molar masses derived via NMR in Supplementary Materials). 1,1′-(methylenedi-4,1-phenylene) bismaleimide (DPBM, 95%) was obtained from Sigma Aldrich (St. Louis, MO, USA). Hydroquinone (1,4-benzenediol, 99%) was used as a radical inhibitor and was supplied by Sigma Aldrich. Chloroform (stab./Amylene) (minimum of 99.9%) was obtained from Biosolve Chimie (Dieuze, France). All chemicals were used as delivered. Figure 2 provides an overview of the monomer units. 

#### 2.1.2. Synthesis

The synthesis of the reversible covalent polymer networks was performed in two steps. First, Jeffamines were functionalized with furan moieties through an irreversible epoxy−amine reaction with monofunctional FGE under stoichiometric conditions without the use of solvent or catalyst. The container was placed in an oil bath at 60 °C and magnetically stirred for 5 days. Then, the temperature was raised to 90 °C for 2 days to complete the epoxy–amine reaction between the amine hydrogens and epoxy functional groups. Secondly, the required amount of Furan, DPBM, and hydroquinone was added to chloroform (20 wt%) in a well-sealed container that was placed on a magnetic stirrer overnight to ensure that all bismaleimide was dissolved in the chloroform. To extract the solvent, the transparent solution was cast in a Teflon mold and kept in a vacuum oven for 24 h. Upon solvent evaporation, the concentration of maleimide and furan increases, pushing the Diels–Alder equilibrium towards the bonded state, resulting in the formation of the polymer network.

#### 2.1.3. Polymer Network Composition

**Figure 3:**
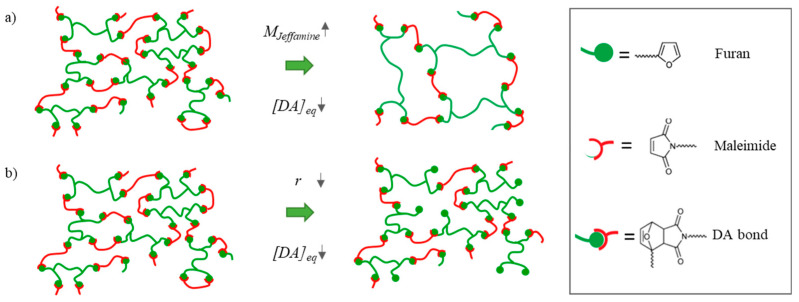
The Diels–Alder crosslink density (*[DA]_eq_*) can be varied using two parameters that were selected in this research: (**a**) By decreasing the molar mass of the furan-containing compound in the synthesis (*M_Jeffamine_*), the crosslink density of the resulting network is reduced. (**b**) By decreasing the off-stoichiometric maleimide-to-furan ratio (*r*), a larger excess of furan is created, resulting in a decrease in crosslink density in the resulting network.

The depicted polymeric networks in Figure 3 are composed of longer aliphatic polypropylene (PPO) oxide chains (shown in green) and shorter aromatic methylene diphenyl chains (depicted in red). These chains are crosslinked by the reversible Diels–Alder reaction. The material properties can be altered by changing the crosslink density in the polymeric network. In this paper, two separate methods are introduced to change the crosslink density and the resulting healing and (thermo-)mechanical performance of the networks. (i) By selecting a Jeffamine with higher molecular weight in the synthesis, the spacer length in between the crosslinks is increased, thereby decreasing the overall crosslink density (Figure 3a). (ii) Alternatively, the crosslink density can be decreased by introducing a deficit of bismaleimide in the network (Figure 3b). This can be controlled via the maleimide-to-furan ratio (*r* = ${\left[M\right]}_{0}/{\left[F\right]}_{0}$) defined as the ratio between the initial maleimide ${\left[M\right]}_{0}$ and furan concentration ${\left[F\right]}_{0}$. When using this method, the spacer length (length of the coiled PPO chain) between the crosslinks is not increased. Instead, dangling chains with unreacted furan are generated in the polymeric network. 

### 2.2. Instruments

#### 2.2.1. Uniaxial Tensile Testing

All materials were characterized by quasistatic uniaxial tensile testing with a fixed strain ramp of 1%.s^−1^ with 0.01 N preload force and 0.01 % initial strain (strain rate mode). This was performed on a TA Instruments DMA Q800 (New Castle, DE, USA) using a film tension clamp. Rectangular tensile samples were used with the following dimensions: 5 × 1 × 30 mm^3^. The gauge length, the initial distance between the clamps, was 5–7 mm. All samples were strained until fracture at ambient conditions, e.g., 25 °C. From these data, the fracture stress and fracture strain were derived, as well as Young’s modulus, which was calculated via linear regression in the 0–15% strain window. The toughness was also calculated by integrating the stress–strain curve for the strain window from zero to the fracture strain. 

#### 2.2.2. Dynamic Mechanical Analysis 

Dynamic mechanical analysis (DMA) was carried out using a TA Instruments DMA Q800 equipped with a liquid nitrogen gas cooling accessory. This technique allows for analyzing the visco-elastic performance of the polymers as a function of temperature from the solid into the near-liquid domain. Again, samples of 5 × 1 × 30 mm^3^ were used. Small amplitude oscillatory measurements were performed to study the viscoelastic properties (storage modulus *E*′, loss modulus *E*″, and the loss angle δ) at a frequency of 1 Hz and 0.1% strain in heat-cool cycles at a rate of 2 K min^−1^ in multi-frequency strain mode, using a track force setting of 115%.

#### 2.2.3. Dynamic Rheometry 

Dynamic rheometry was performed with a TA Instruments Discovery Hybrid Rheometer (DHR2) to determine the (de)gelation temperature. This technique allows for analyzing the visco-elastic performance of the polymers as a function of temperature from the solid into the liquid domain. The experiment was performed using a 15 mm aluminum parallel plates geometry in a temperature range from 60 °C to 140 °C and a heating rate of 1 K min^−1^, which was subjected to an oscillatory strain with an amplitude of 5% at different frequencies: 0.312, 0.562, 1.0, 1.778, and 3.125 Hz. 

#### 2.2.4. Differential Scanning Calorimetry

Differential scanning calorimetry (DSC) analyses were conducted utilizing a TA Instruments Discovery DSC. The DSC was furnished with a refrigerated cooling system (RCS) that enabled cooling down to −90 °C. Purge gas, specifically nitrogen, was employed. The Diels–Alder network samples, weighing between 15 and 20 mg, were assessed in TA Instruments Tzero pans fitted with perforated Tzero hermetic lids to maintain an inert atmosphere over the sample. The thermal behavior was tested by subjecting the samples to a temperature ramp of 10 K min^−1^, ranging from −90 °C to 130 °C.

#### 2.2.5. Thermogravimetric Analysis 

Thermogravimetric Analysis (TGA) was performed on a TA instrument Q5000IR with an auto-sampler. Nitrogen was used as a purge gas in order to perform the experiment in inert conditions. The samples (2–5 mg) were placed in 100 µL platinum crucibles. The samples were subjected to heating at a ramp rate of 10 K min^−1^, spanning from 60 °C to 600 °C, with concurrent tracking of the relative weight alteration

### 2.3. Methods

#### 2.3.1. Self-Healing and Healing Efficiency

**Figure 4:**
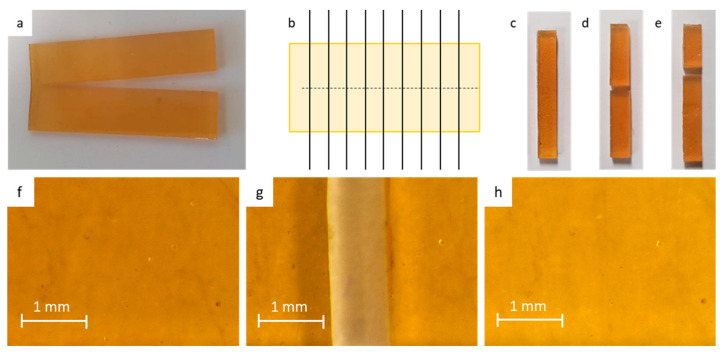
Preparation and testing of the healed tensile samples: (**a**) 60 × 30 × 1 mm^3^ sheets are cut in half using a razor blade. Immediately after cutting, the fracture surfaces are brought back into contact. (**b**) The rectangular 5 × 30 × 1 mm^3^ are cut. (**c**) The tensile samples are left to heal at room temperature. (**d**) Upon fracturing a sample that was not completely healed due to insufficient healing time, the sample fractures at the location of the scar. (**e**) Given enough time at room temperature, the fracture occurs at a location that is different from that of the scar. This illustrates full recovery of the mechanical properties. (**f**–**h**) Microscopic images of the samples prior to damage (**f**), after damage (**g**), and seconds after recontact (**h**).

In this paper, we focused on room temperature healing of large macroscopic damages, e.g., cutting a tensile sample completely in half using a scalpel blade. After this large damage, the fracture surfaces are reconnected almost instantly in order to prevent the recombination of the reactive furan and maleimide into Diels–Alder bonds in the separated fracture surfaces. To minimize notches due to misalignment, e.g., geometrical defects, large sheets were cut and healed prior to separating the sheet into tensile samples of 5 × 1 × 30 mm^3^ (Figure 4). The samples were left to heal at room temperature for different healing times and were subsequently fractured in the tensile test. To illustrate excellent sealing after recontact, microscopic images were made on the DPBM-FT5000-r0.5 material prior to the damage, after damage, and seconds after recontact (Figure 4f–h). From the images, it is evident that upon recontact, the seal is nearly flawless, displaying only a minimal scar. This surface-level imperfection arises due to geometric discrepancies and, regrettably, does not self-repair over the course of hours or days. However, it is important to note that despite its limited aesthetic impact, this scar does not detract from the overall effectiveness of the healing process, as demonstrated in the subsequent experimental section.

One of the most common methods for analyzing self-healing is healing efficiency. The healing efficiency ($\eta_{p}$), based on the property (*p*), can be defined by the following Equation (1).

(1) $$\eta_{p}=\frac{p_{healed}-p_{damaged}}{p_{initial}-p_{damaged}}$$

In this equation, the property (*p*) can be a mechanical property, including Young’s Modulus (*E*), fracture stress (*σ_max_*), fracture strain (ε*_max_*), or an electrical property, including resistance (*R*) or conductivity (*σ*) or another. In this paper, the healing was investigated on samples that were cut completely in half by a scalpel and brought back in contact based on the recovery of *E*, *σ_max_,* and ε*_max_*. All properties are zero in the damaged samples that are fully cut. Therefore, Equation (1) for the healing efficiency is reduced to Equation (2):

(2) $$\eta_{p}=\frac{p_{healed}}{p_{initial}}$$

#### 2.3.2. Kinetics Simulation

The Diels–Alder reaction is described by the reaction of maleimide (M) and furan (F) into two isomers of the Diels–Alder Adduct, e.g., an exo and endo isomer [33]. These two equilibrium reactions are defined by four reaction rates (*k*) that are a function of temperature and given by an Arrhenius Equation (3):

(3) $$k_{DA,i}=A_{DA,i}e^{\frac{-E_{DA,i}}{RT}}\;\;\;\;\;\;\;\;\;\;\;\;\;\;\;\;\;\;k_{rDA,i}=A_{rDA,i}e^{\frac{-E_{rDA,i}}{RT}}\;$$

where “*i*” indicates endo or exo, *A_DA,i_* and *A_rDA,i_* are the pre-exponential factors or frequency factors, and *E_DA,i_* and *E_rDA,i_* are the activation energies. *R* is the universal gas constant, and *T* is the absolute temperature in Kelvin. The kinetic parameters (*A_DA,i_, A_rDA,i_, E_DA,i_,* and *E_rDA,i_*) were derived from experimental heat flow measurements performed in DSC [21]. Based on these reaction rate constants, the maleimide *[M]*, furan *[F],* and Diels–Alder (*[DA] = [DA_,exo_] + [DA_,endo_]*) concentrations can be calculated for a given time and temperature by the set of ordinary differential equations (ODEs), presented in Equation (4):

(4) $$\begin{array}{cc} & \frac{d\left[M\right]}{dt}=\frac{d\left[F\right]}{dt}=\sum_{i=exo,endo}\left\{{\;k_{rDA,i}\left[DA,i\right]-k}_{DA,i}\left[M\right]\left[F\right]\;\right\}=-\frac{d\left\{\;\left[DA,exo\right]+\left[DA,endo\right]\right\}}{dt}=-\frac{d\left[DA\right]}{dt}\;\;\\ & \frac{d\left[DA,i\right]}{dt}=\;k_{DA,i}\left[M\right]\left[F\right]-\;k_{rDA,i}\left[{DA}_{i}\right]\;\;\; \end{array}$$

Next, the (overall) reaction conversion or extent of the reaction *x* is defined by Equation (5):

(5) $$x=\frac{\left[DA,exo\right]+\left[DA,endo\right]}{{\left[M\right]}_{0}}=\frac{\left[DA\right]}{{\left[M\right]}_{0}}$$

For a given temperature, the conversion at equilibrium $x_{eq}$ can be calculated using the dimensionless concentration-based equilibrium constants ($K_{C,\;exo}$ and $K_{C,\;endo}$) and *C_*0*_* a standard concentration of 1 mol.kg^−1^, which are given in Equation (6):

(6) $$\begin{array}{cc} & K_{C,endo}=\frac{k_{DA,endo}C_{0}}{k_{rDA,endo}}=\frac{{\left[{DA}_{endo}\right]}_{eq}C_{0}}{{\left[F\right]}_{eq}{\left[M\right]}_{eq}}\\ & K_{C,exo}=\frac{k_{DA,exo}C_{0}}{k_{rDA,exo}}=\frac{{\left[{DA}_{exo}\right]}_{eq}C_{0}}{{\left[F\right]}_{eq}{\left[M\right]}_{eq}}\\ & {K_{C,DA}=K}_{C,\;endo}+K_{C,\;exo}=\frac{{\left[{DA}_{endo}\right]}_{eq}C_{0}+{\left[{DA}_{exo}\right]}_{eq}C_{0}}{{\left[F\right]}_{eq}{\left[M\right]}_{eq}}=\frac{x_{eq}C_{0}}{(\frac{1}{r}-x_{eq})(1-x_{eq}){\left[M\right]}_{0}} \end{array}$$

Consequently, the equilibrium conversion $x_{eq}$ can be derived and expressed by Equation (7).

(7) $$x_{eq}=\;\frac{K_{C,DA}(1+\frac{1}{r}){\left[M\right]}_{0}+1-\sqrt{{\left(K_{C,DA}(1+\frac{1}{r}){\left[M\right]}_{0}+1\right)}^{2}-\frac{4{K_{C,DA}}^{2}{{\left[M\right]}_{0}}^{2}}{r}}}{2K_{C,DA}{\left[M\right]}_{0}(\frac{1}{C_{0}})}$$

Upon heating, at the degelation transition, the polymer network breaks down into a viscous polymer due to the extensive breaking of the Diels–Alder crosslinks. This transition is reversible, as upon cooling, the network is reformed due to Diels–Alder binding. The (de)gelation conversion (*x_gel_*) can be defined by the Flory Stockmayer Equation (8) [34].

(8) $$x_{gel}=\;\frac{1}{\sqrt{r\left(f_{M}-1\right)(f_{F}-1)}}$$

## 3. Results

### 3.1. Varying the Diels–Alder Concentration through the Molecular Weight of the Monomers 

The Diels–Alder concentration at room temperature in the reversible networks can be altered by changing the molecular weight of the monomers used in the synthesis as well as by changing the maleimide to furan ratio r. In this first section, the influence of the Diels–Alder concentration on the healing and mechanical performance was investigated through four Diels–Alder polymer networks: two triamine-based networks, DPBM-FT3000-r0.5 and DPBM-FT5000-r0.5, as well as two diamine-based networks, DPBM-FD2000-r0.6 and DPBM-FD4000-r0.6. These have equal stoichiometric ratio *r* and functionality *f* but differ in Diels–Alder concentration due to the use of a furan functionalized compound (FTxxx and FDxxx) with a different molecular mass. The information on their network composition is summarized in Table 1. The equilibrium Diels–Alder, furan, and maleimide concentrations at room temperature were calculated via Equation (7). 

#### 3.1.1. Quasistatic Mechanical Performance at Room Temperature

**Figure 5:**
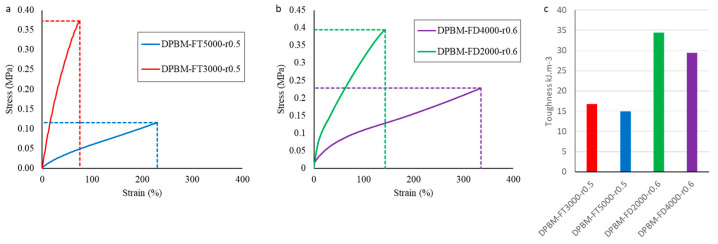
Uniaxial tensile test until fracture with a fixed strain ramp of 1%.s^−1^ for the triamine-based elastomers (**a**) and the diamine-based elastomers (**b**). In (**c**), the resulting toughness’s are presented, which were calculated by integrating the stress–strain curve in the total strain window until fracture.

The mechanical properties are first investigated by uniaxial tensile testing, in which rectangular samples (5 × 1 × 30 mm^3^) are strained until fracture with a contact strain of 1%.s^−1^ (Figure 5a,b). From these tests, it is clear that decreasing the Diels–Alder concentration leads to an increase in flexibility illustrated by larger fracture strains, lower fracture stresses, and lower moduli for both the diamine- and triamine-based polymer networks. It is also clear that although the Diels–Alder concentration of DPBM-FD4000-r0.6 and DPBM-FT5000-r0.5 are close, the diamine-based network can withstand much larger strains before fracture. DPBM-FD4000-r0.6 reaches up to strains of 330%, while DPBM-FT5000-r0.5 strains only reach up to 240%. This is due to the fact that the diamine-based network, the DPBM-FD4000-r0.6, has a higher stoichiometric ratio. Therefore, for similar Diels–Alder concentrations, it contains a lower number of dangling chains. Consequently, the coiled chain segments between the Diels–Alder crosslinks are longer and can be stretched to higher strains. This is emphasized by a higher toughness in these materials, which were calculated by calculating the integral of the stress–strain curve in the appropriate strain window (0% fracture strain) (Figure 5c). In addition, it can be seen that a change in the crosslink density also slightly decreases the toughness of the material.

#### 3.1.2. Self-Healing Performance at Room Temperature

**Figure 6:**
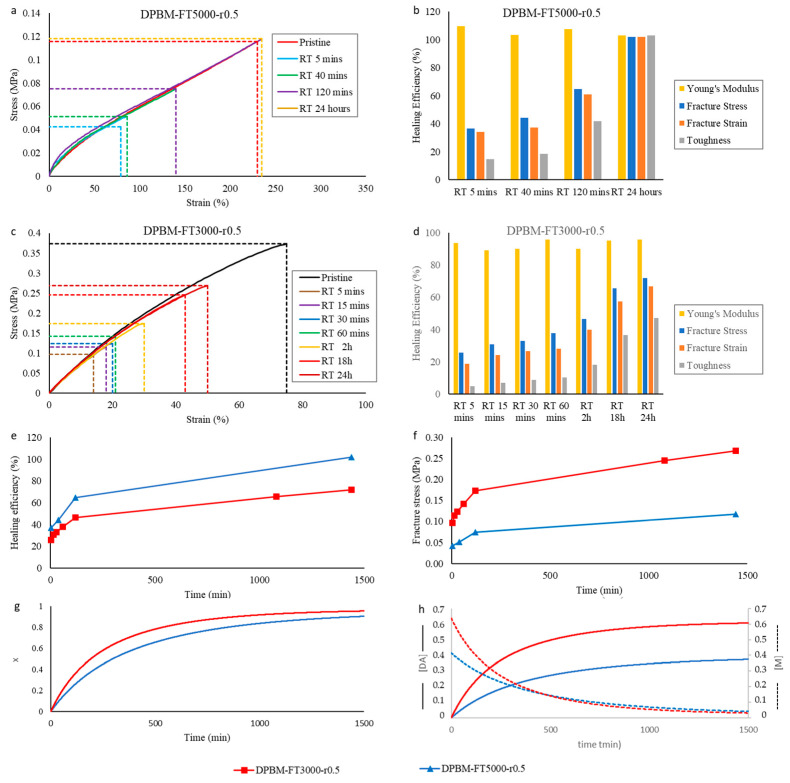
Healing tests based on uniaxial tensile testing of healed samples and reaction simulations for the triamine-based samples: (**a**,**c**) Evolution of the stress as function of strain during the uniaxial tensile test with fixed strain ramp of 1%.s^−1^. The pristine, undamaged samples are compared with samples that were healed at different times. (**b**,**d**) The resulting healing efficiencies are based on the recovery of Young’s modulus, fracture stress, fracture strain, and toughness. (**e**,**f**) Comparison of the increase in healing efficiency (**e**) and fracture stress (**f**) as a function of time for the two triamine-based networks with different crosslink densities. (**g**,**h**) Comparison of the increase in simulated conversion *x* (**g**), Diels–Alder concentration *[DA],* and maleimide concentration *[M]* (**h**) as a function of time for the two triamine-based networks with different crosslink densities.

In order to investigate the self-healing, tensile samples (5 × 1 × 30 mm^3^) were cut in half using a scalpel blade and brought back in contact immediately. Next, the samples were left to cure at 25 °C for different times, up to 24 h, after which they were tested until fracture in the tensile test with a strain rate of 1%.s^−1^. The results are compared with the tensile curves of the pristine samples and are presented in Figure 6a,c.

First, it can be seen that after only 5 min of healing time, part of the mechanical properties are already recovered. For DPBM-FT5000-r0.5, the sample can even be strained up to 79%. It fractures at the location of the scar with a clean fracture (Figure 4d). In this strain window (0–75%), the stress–strain relation is recovered, as the curve of the healed sample is nearly identical to the one of the pristine sample. This is confirmed by a near 100% healing efficiency based on the recovery of Young’s modulus for this very short healing time (Figure 6b). This instantaneous healing, also visible in Figure 1, results from reactive maleimide and furan at the fracture surfaces that were formed upon mechanical breaking of the Diels–Alder bonds (cutting). When bringing the fracture surfaces back in contact, these reactive groups are very eager to react. In addition, other adhesive forces and secondary interactions assist this first healing. The effect of these non-covalent interactions is higher for the elastomer with lower Diels–Alder concentration, the DPBM-FT5000-r0.5, as their contribution is higher relative to the number of Diels–Alder bonds. 

Next, it is clear that the mechanical properties are further recovered upon increasing the healing time. When observing the healing efficiency Figure 6b, defined as the recovery of Young’s modulus, fracture stress, fracture strain, or toughness, it can be concluded that the DPBM-FT5000-r0.5 elastomer recovers full performance after 24 h at room temperature. After this time, the fracture does not occur at the location of the scar but at another site (Figure 4e). This is another indicator of the full recovery of the mechanical properties as the sample now breaks at a location where stress concentrations are present due to the clamping and/or due to a small defect, e.g., a dust particle or an air bubble. Although Diels–Alder-based self-healing polymers are considered to be non-autonomously self-healing polymers in the literature, it is illustrated here that they can heal fast at ambient conditions. 

The healing process can be simulated by the increase in Diels–Alder concentrations and/or Diels–Alder conversion as a function of time (Figure 6g). In this simulation, it is assumed that at the fracture surfaces, all Diels–Alder bonds are cut; therefore, the Diels–Alder concentration and the conversion are zero. Consequently, the maleimide and furan concentrations are equal to the initial concentrations, [*M*_0_] and [*F*_0_], given in Table 1. After damage, the fracture surfaces are immediately brought back together. During healing, the theoretical Diels–Alder concentration *[DA]* (Figure 6g) and the conversion *x* (Figure 6h) increase as a function of time following the differential Equation (4). 

However, autonomous healing comes with a drawback: the fracture surfaces must be promptly reconnected after healing. If the damage remains exposed for an extended period, the reactive furan and maleimide groups generated by the damage will undergo separate recombination at the fracture surfaces. As a result, when the fracture surfaces are brought back into contact, there are no remaining reactive components, rendering healing impossible within a reasonable timeframe (e.g., less than one day). Figure 6h provides a simulation demonstrating the presence of maleimide groups on open fracture surfaces. It is evident that the concentration of reactive maleimide groups rapidly diminishes over time, thereby losing the ability to facilitate healing.

Upon comparing the DPBM-FT5000-r0.5 and DPBM-FT3000-r0.5, it can be noticed that the recovery of the mechanical properties is faster for the network with a lower Diels–Alder crosslink density. This can be highlighted by the increase in the healing efficiency based on the recovery of the fracture stress as a function of time, which is faster for DPBM-FT5000-r0.5 (Figure 6e). Upon looking at the speed of recovery of the Diels–Alder concentration, illustrated by the simulated conversion x in Figure 6g, it can be seen that conversion increases faster for the higher crosslinked DPBM-FT3000-r0.5 material. When taking only the speed of reaction into account, networks with a higher Diels–Alder crosslink density should heal faster. However, experimentally, in the recovery of the mechanical properties, the opposite is observed. This illustrates that besides reaction kinetics, mobility also plays a crucial role. Decreasing the Diels–Alder concentration using monomers with lower molecular weight leads to an increase in mobility, expressed by a lower Young’s modulus (Figure 5a).

Although the healing efficiency increases faster for the lower crosslinked material, the fracture stress is higher during the healing process for the higher crosslinked material (Figure 6f). Therefore, although it has lower healing efficiencies, the higher crosslinked material can withstand higher stresses at a faster rate. Depending on the application, being able to withstand forces or stresses faster can be prioritized over the recovery of the initial properties, e.g., high healing efficiencies. This is confirmed by the simulation of the increase in Diels–Alder concentrations as a function of time during the healing procedure (Figure 6h). As more Diels–Alder crosslinks are formed as a function of time for higher crosslinked networks, it takes more stress to break the reformed bonds across the fracture surfaces. 

**Figure 7:**
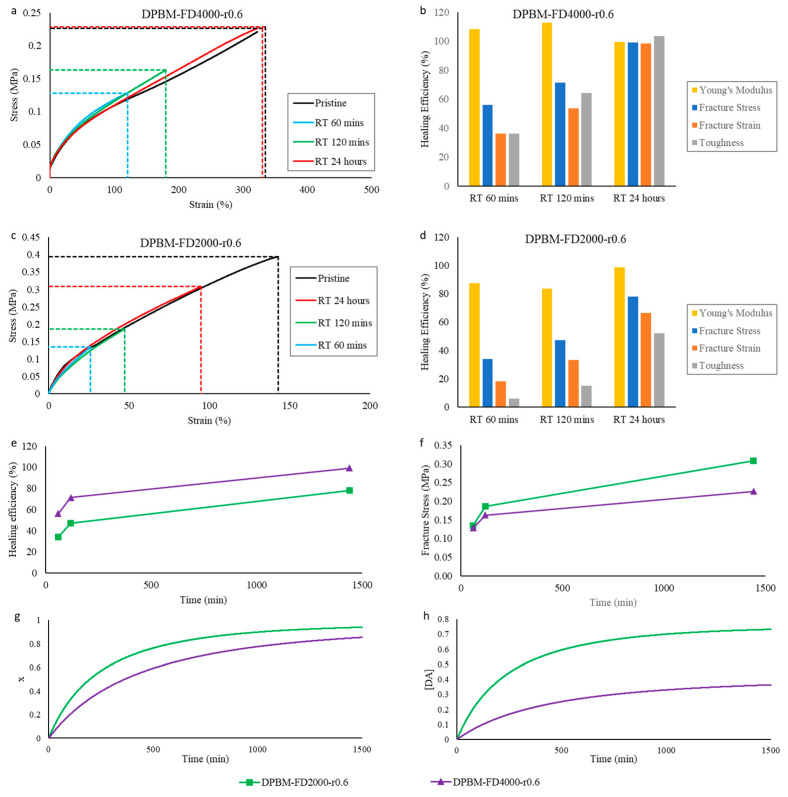
Healing tests based on uniaxial tensile testing of healed samples and reaction simulations for the diamine-based Diels–Alder polymers: (**a**,**c**) Evolution of the stress as function of strain during the uniaxial tensile test with fixed strain ramp of 1%.s^−1^. The pristine, undamaged samples are compared with samples that were healed at different times. (**b**,**d**) The resulting healing efficiencies based on the recovery of Young’s modulus, fracture stress, fracture strain and toughness. (**e**,**f**) Comparison of the increase in healing efficiency (**e**) and fracture stress (**f**) as a function of time for the two diamine-based networks with different crosslink densities. (**g**,**h**) Comparison of the increase in simulated conversion *x* (**g**) and Diels–Alder concentration *[DA]* (**f**) as a function of time for the two diamine-based networks with different crosslink densities.

In view of reproducibility, the above experiments were repeated for the two diamine-based networks, DPBM-FD4000-r0.6 and DPBM-FD2000-r0.6 (Figure 7). In these experiments, all the above-mentioned observations were confirmed. 

#### 3.1.3. Visco-Elastic Behaviour as Function of Temperature

**Figure 8:**
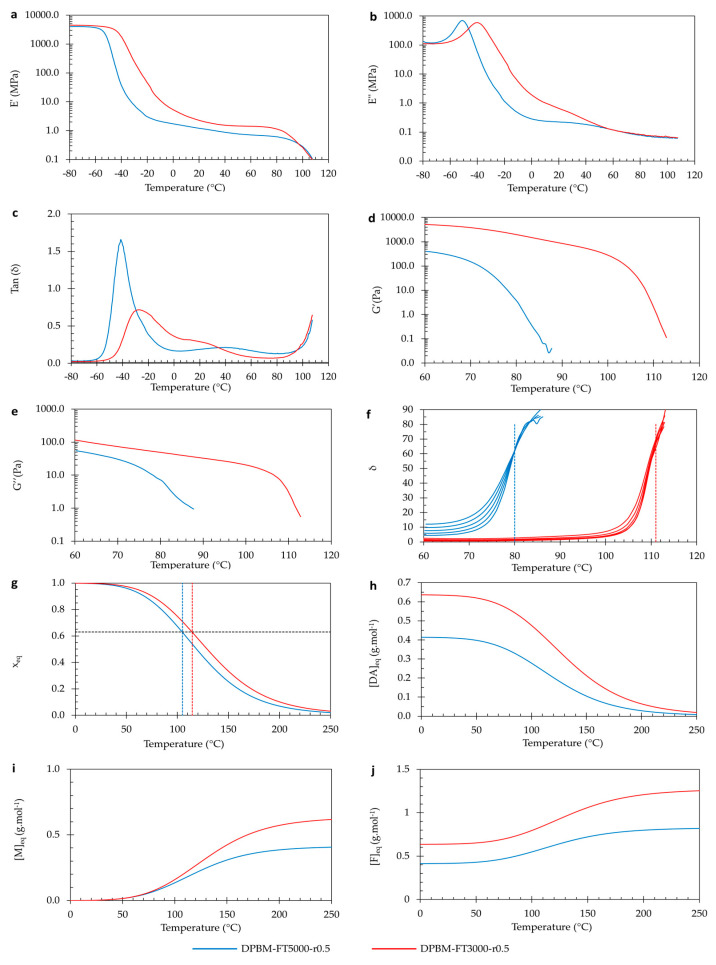
Visco-elastic behavior as a function of temperature of the triamine-based Diels–Alder polymers: (**a**–**c**) Dynamic mechanical analysis (DMA) as a function of temperature, presented by the storage modulus (**a**), loss modulus (**b**), and Tan delta (**c**). (**d**–**f**) Dynamic rheometry as a function of temperature, presented by the storage modulus (**d**), loss modulus (**e**), and phase angle (**f**). To illustrate the degelation temperature, the loss angle as a function of temperature is plotted for different frequencies 3.1 Hz, 1.8 Hz, 1 Hz, 0.6 Hz, and 0.3 Hz. (**g**–**j**) Simulation of the equilibrium conversion x_eq_ (**g**), the Diels–Alder concentration *[DA*_eq_*]* (**h**), the maleimide concentration *[M*_eq_*]* (**i**), and the furan concentration *[F*_eq_*]* as a function of temperature.

The Diels–Alder polymers are thermo-reversible, meaning their Diels–Alder crosslinks can also be reversibly broken via thermal treatment. This property is experimentally illustrated by studying their visco-elastic behavior as a function of temperature in DMA and dynamic rheometry (Figure 8a–f). First, in DMA, the solid visco-elastic behavior is investigated (Figure 8a–c). Upon heating from −60 °C, the storage modulus (*E*′) decreases, while the loss modulus (*E*″) increases, eventually reaching a maximum. The maximum in *E*″ reflects the glass transition temperature (*T_g_*). At this temperature, the polymer network’s mechanical behavior changes from a stiff thermoset behavior (energy elasticity) into flexible elastomeric behavior (entropy elasticity). This transition can also be observed by looking at maxima of the loss angles (*δ*), which is the arctangent of the ratio between *E*′ and *E*″ (Figure 8c). It is clear that higher crosslinked networks, like the DPBM-FT3000-r0.5, have a higher *T_g_*. This results from a decrease in molecular mobility in the network due to a higher concentration of Diels–Alder crosslinks. Above *T_g_*, both *E*′ and *E*″ further decrease due to a decrease in Diels–Alder crosslinks, leading to an increase in flexibility.

The visco-elastic behavior as a function of temperature can be further investigated in the viscous domain through dynamic rheometry (Figure 8d–f). Upon heating to higher temperatures, the polymers are softened. Upon reaching the degelation temperature, the polymers experience a fast decrease in both moduli. However, the storage modulus (*G*′) decreases relatively faster than the loss modulus (*G*″), illustrating that the contribution of the viscous compound of the visco-elastic behavior increases. This is emphasized by looking at the loss angle (*δ*). An angle of 45° coincides with the temperature at which the storage and loss moduli intersect. Although this temperature is often defined as the experimental (de)gelation temperature (*T_gel_*), in this paper, *T_gel_* is defined according to [35] as the temperature at which the loss angle (*δ*) becomes frequency independent. Figure 8f shows the relation between *δ* and temperature for different frequencies for the two networks, illustrating the intersection point and the related experimental *T_gel_*’s. The DPBM-FT5000-r0.5 has a *T_gel_* of 80 °C, while the DPBM-FT3000-r0.5 has a *T_gel_* of 112 °C. Higher Diels–Alder concentrations lead to higher *T_gel_*s. Consequently, the (re)processing temperatures of these materials are higher. It can also be observed that degelation at higher temperatures leads to sharper transitions, e.g., in a more narrow temperature window. This results from the higher reaction rates (*k*) at higher temperatures in Equation (3).

The change in Diels–Alder concentration as a function of temperature and resulting concentration *x_eq_* can be simulated using the equilibrium equation in Equation (7). This is presented in Figure 8g,h, along with the maleimide *[M]_eq_* and furan concentrations *[F]_eq_* in Figure 8i,j. At low temperatures, the conversion (*x*) is near one, indicating that almost all maleimide present in the network is reacted. Next, it is clear that the Diels–Alder concentration decreases with increasing temperature, creating more reactive maleimide and furan in the network. The conversion *x_eq_* of the DPBM-FT5000-r0.5 drops faster than the DPBM-FT3000-r0.5. The degelation temperature can be simulated by the intersection between the equilibrium conversion *x_eq_* and the (de)gelation conversion *x_gel_*. In other words, by solving the system of Equations (7) and (8) towards temperature *T*, *T_gel_* is gained. The simulated *T_gel_*s of the DPBM-FT5000-r0.5 and DPBM-FT3000-r0.3 are 104 °C and 114 °C, respectively. Although the simulation shows a similar trend as the experimental *T_gel_*, the simulated values are higher. 

**Figure 9:**
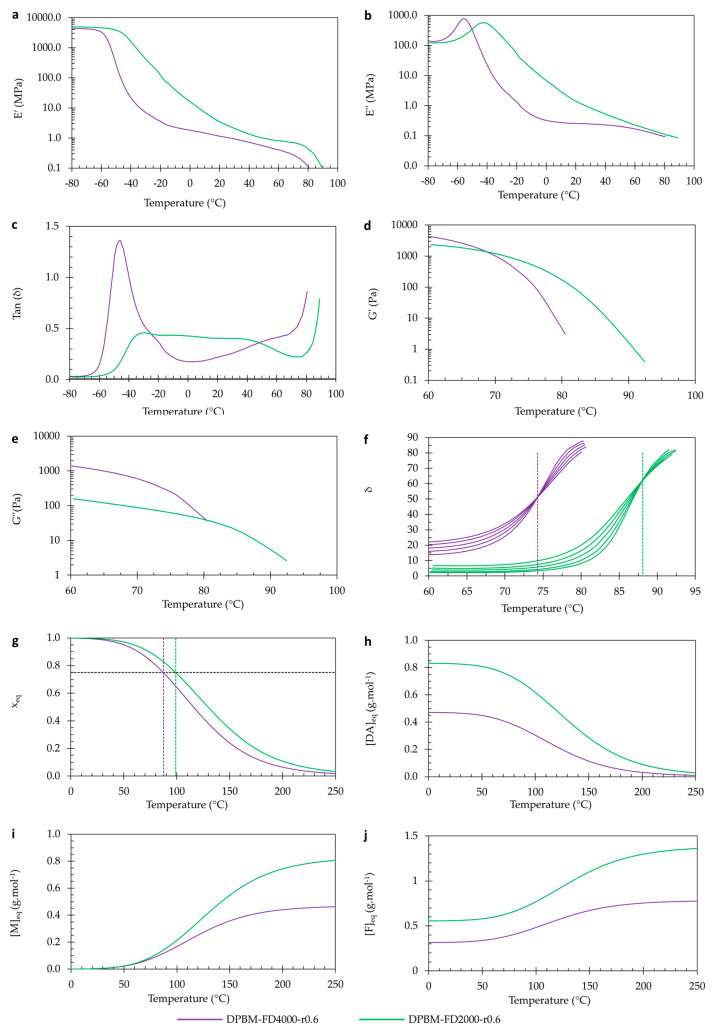
Visco-elastic behavior as a function of temperature of the diamine-based Diels–Alder polymers: (**a**–**c**) Dynamic mechanical analysis (DMA) as a function of temperature, presented by the storage modulus (**a**), loss modulus (**b**), and loss angle (**c**). (**d**–**f**) Dynamic rheometry as a function of temperature, presented by the storage modulus (**d**), loss modulus (**e**), and loss angle (**f**). To illustrate the degelation temperature, the loss angle as a function of temperature is plotted for different frequencies 3.1 Hz, 1.8 Hz, 1 Hz, 0.6 Hz, and 0.3 Hz. (**g**–**j**) Simulation of the equilibrium conversion x_eq_ (**g**), the Diels–Alder concentration *[DA*_eq_*]* (**h**), the maleimide concentration *[M*_eq_*]* (**i**), and the furan concentration *[F*_eq_*]* as a function of temperature.

To double-check the reproducibility of these results, the diamine-based polymers were investigated using the same conditions (Figure 9). Very similar observations were made by comparing the two diamine-based polymer networks.

#### 3.1.4. Thermal Behaviour as Function of Temperature

**Figure 10:**
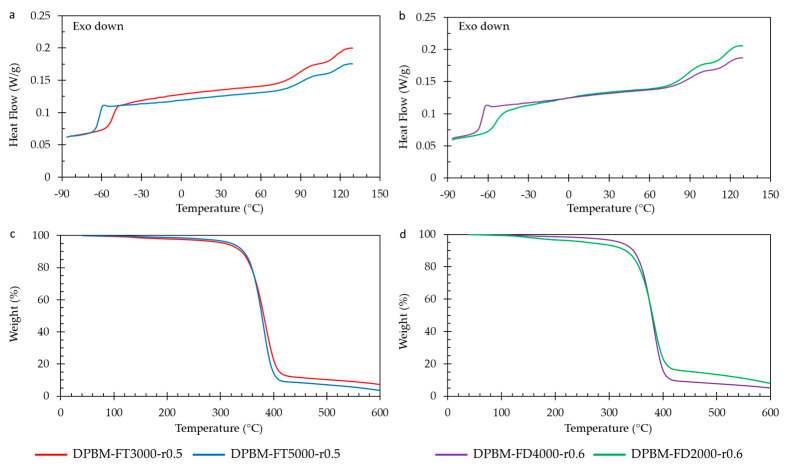
Differential scanning calorimetry (DSC) and thermogravimetric analysis (TGA) for the diamine and triamine-based Diels–Alder polymers: (**a**) DSC of the triamine-based polymers. (**b**) DSC of the diamine-based polymers. (**c**) TGA of the triamine-based polymers. (**d**) TGA of the diamine-based polymers.

The thermal characteristics of the polymer networks are examined using differential scanning calorimetry (DSC) and thermogravimetry (TGA), with the corresponding results presented in Figure 10. DSC reveals the glass transition temperature (*T_g_*), represented by a sudden rise in the heat flow signal. This increase in heat capacity results from enhanced mobility during devitrification. When comparing both DSC analyses (Figure 10a,b), it is evident that *T_g_* escalates with an increase in Diels–Alder concentration. An increase in Diels–Alder concentration due to the lower molecular weight of the monomers leads to reduced mobility in the amorphous network. This restricted mobility leads to a glass transition at higher temperatures.

At higher temperatures (80–130 °C), all networks exhibit a double peak phenomenon, with peaks occurring all at 95 °C and 125 °C. This phenomenon arises from the retro-Diels–Alder reaction, specifically the detachment of reversible bonds. The two peaks correspond to the exo and endo retro-Diels–Alder reactions. The first peak represents the kinetically favored endo isomer, while the second peak results from the thermodynamically more stable exo isomer. Networks with higher Diels–Alder concentration exhibit a more pronounced endothermic peak due to a larger quantity of Diels–Alder bonds that can be disrupted during temperature elevation, resulting in greater heat absorption.

Thermal stability assessment of the Diels–Alder networks was conducted using TGA (Figure 10c,d). The samples underwent heating with a ramp rate of 10 K min^−1^, ranging from 60 °C to 600 °C while monitoring the relative weight change. Within the temperature range from 120 °C to 300 °C, a slight decrease in a few weight percent (±5%) was observed. This decrease is attributed to the liberation of small amounts of trapped chloroform (CHCl_3_) during network synthesis at higher temperatures. Notably, the authors have developed a solventless synthesis method for these materials [36]. All networks exhibited degradation starting from 300 °C, with the furan-functionalized Jeffamine undergoing degradation and the escape of volatile compounds, leading to a reduction in sample weight. The maleimide compound, particularly the aromatic variant, displayed higher stability and degraded at higher temperatures. When comparing the materials, networks with a higher maleimide content showed marginally higher weight percentages after the initial degradation step. These degradation temperatures provide significant characterization insights for the DA networks. However, it is crucial to note that they should not be considered critical temperatures for applications and reprocessing due to the occurrence of homopolymerization of maleimide at elevated temperatures (150–160 °C) [37,38]. This irreversible crosslinking results in a loss of reprocessability and healing capacity.

### 3.2. Varying the Diels–Alder Concentration through the Stoichiometric Ratio

Next, the Diels–Alder concentration varied by changing the ratio between the furan and maleimide-containing monomer compounds. Three Diels–Alder networks, DPBM-FT3000-r0.5, DPBM-FT3000-r0.4, and DPBM-FT3000-r0.3, which differ in maleimide-to-furan ratio *r*, were compared. The information on their network composition is summarized in Table 2. The equilibrium Diels–Alder, furan, and maleimide concentrations at room temperature were again calculated via Equation (7).

#### 3.2.1. Quasistatic Mechanical Performance at Room Temperature

**Figure 11:**
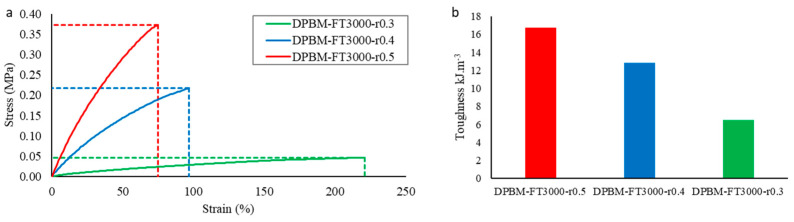
(**a**) Uniaxial tensile test until fracture with a fixed strain ramp of 1%.s^−1^ for the triamine-based elastomers with varying maleimide-to-furan ratio *r*. In (**b**), the resulting toughness’s are presented, which were calculated by integrating the stress–strain curve in the total strain window until fracture.

For each of the three triamine-based networks, the mechanical performance was again first measured using uniaxial tensile testing (Figure 11a). The toughness that was calculated from these graphs is presented in Figure 11b. As the crosslink density decreases for decreasing maleimide-to-furan ratio *r*, Young’s modulus and the fracture stress decrease upon decreasing *r*, while the strain at fracture increases. When comparing these results with the one in Figure 5b, it can be observed that decreasing the crosslink density using the r parameter leads to a faster decrease in the toughness of the material. The reason is that, upon lowering the *r*-ratio, the number of dangling chains is increased while the length of the coiled chain segment in between the Diels–Alder crosslinks remains the same. This is in contrast to lowering the crosslink density by changing the molecular weight of the monomers, which increases the length of the coiled chain segment. Therefore the flexible networks presented in Figure 11 cannot be strained as much as the ones in Figure 5a, hence lowering the toughness of the materials. 

#### 3.2.2. Self-Healing Performance at Room Temperature

**Figure 12:**
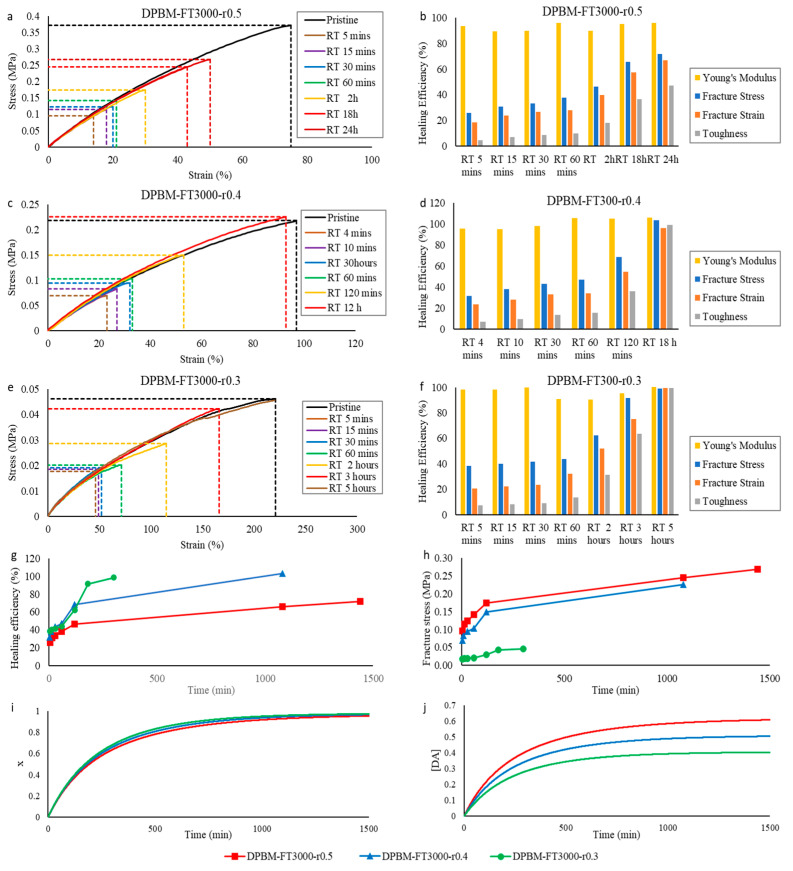
Healing tests based on uniaxial tensile testing of healed samples and reaction simulations for the triamine-based samples: (**a**,**c**,**e**) Evolution of the stress as a function of strain during the uniaxial tensile test with fixed strain ramp of 1%.s^−1^. The pristine, undamaged samples are compared with samples that were healed at different times. (**b**,**d**,**f**) The resulting healing efficiencies are based on the recovery of Young’s modulus, fracture stress, fracture strain, and toughness. (**g**,**h**) Comparison of the increase in healing efficiency (**g**) and fracture stress (**h**) as a function of time for the two triamine-based networks with different crosslink densities. (**i**,**j**) Comparison of the increase in simulated conversion *x* (**i)** and Diels–Alder concentration *[DA]* (**j**) as a function of time for the two triamine-based networks with different crosslink densities.

The healing at room temperature was tested for each of the triamine-based networks with different *r*-ratio. The results of the tensile tests are presented in Figure 11. Also, in this series, we observe immediate healing that is assisted by secondary interactions, illustrated by a fast increase in healing efficiency at the very start of the healing procedure, which is flattened for larger healing times (Figure 12g). It is observed that by lowering the *r*-ratio, the healing speed can be drastically increased. This is illustrated by the DPBM-FT3000-r0.5, which recovers full performance by healing at room temperature for 12 h, while DPBM-FT3000-r0.3 only needs 5 h to completely recover its initial properties. The reason for this can be explained by the speed of the reaction, which is simulated in Figure 12i. The conversion x increases faster for lower *r*-ratio materials. This is caused by the increase in excess furan in the lower *r*-ratio materials (Table 2). For a higher excess of furan, the speed of reaction is increased according to Equation (4). In addition, lowering the *r*-ratio also increases network mobility. Consequently, decreasing the r-ratio leads to both an increase in reaction speed, as well as in mobility, leading to a significant increase in healing performance. Nevertheless, as for the previous series, a faster increase in healing efficiency does not mean that the material can faster withstand higher stresses. This is illustrated experimentally by looking at the increase in fracture stress as a function of healing time (Figure 12h) and in simulation by calculating the Diels–Alder concentration as a function of the healing time (Figure 12j). The networks with a higher *r*-ratio can withstand faster larger stresses due to their higher number of bonds at the fracture site. 

#### 3.2.3. Visco-Elastic Behaviour as Function of Temperature

**Figure 13:**
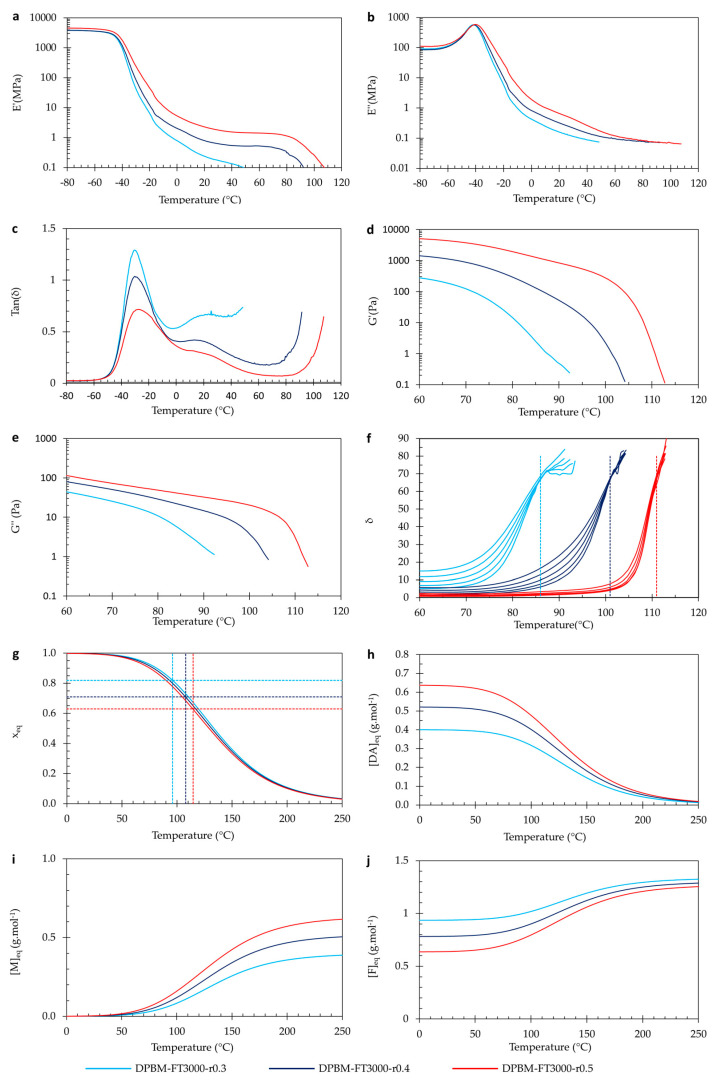
Visco-elastic behavior as a function of temperature of the triamine-based Diels–Alder polymers with varying *r*-ratio: (**a**–**c**) Dynamic mechanical analysis (DMA) as a function of temperature, presented by the storage modulus (**a**), loss modulus (**b**), and loss angle (**c**). (**d**–**f**) Dynamic rheometry as a function of temperature, presented by the storage modulus (**d**), loss modulus (**e**), and loss angle (**f**). To illustrate the degelation temperature, the loss angle as a function of temperature is plotted for different frequencies 3.1 Hz, 1.8 Hz, 1 Hz, 0.6 Hz, and 0.3 Hz. (**g**–**j**) Simulation of the equilibrium conversion *x_eq_* (**g**), the Diels–Alder concentration *[DA*_eq_*]* (**h**), the maleimide concentration *[M*_eq_*]* (**i**), and the furan concentration *[F*_eq_*]* as a function of temperature.

Also, for the polymer networks with varying *r*-ratio, the visco-elastic properties were investigated as a function of temperature (Figure 13). When observing DMA in Figure 12a–c, it can be seen that the glass transition (*T_g_*), seen as a maximum in the loss modulus (*E*″) signal, is slightly changed upon changing the *r*-ratio. The difference is much less pronounced than when decreasing the Diels–Alder concentration using the monomer molecular weight. Upon decreasing concentration by using longer monomeric chains, the spacer length between the crosslinks is increased (Figure 3). This increase in flexibility leads to vitrification of the mobility at lower *T_g_*s. Upon decreasing the concentration by the r-ratio, the spacer length between the crosslinks is not increased. Instead, dangling chains are formed (Figure 3). Therefore, the dangling chains restrict mobility. Nevertheless, the mobility is slightly increased due to a decrease in crosslink density. Due to these counteracting phenomena, the decrease in *T_g_* is limited. Upon increasing the temperature further, we see the same observations as discussed in Section 3.1.3.

By analyzing the viscoelastic properties at room temperature (25 °C), it is evident that reducing the r-ratio causes a decline in both *E*′ and *E*″. The *tan(δ)* ratio, which represents the relationship between these moduli, demonstrates that the network with the lowest *r*-value exhibits the highest *tan(δ)*. In simpler terms, this polymer displays the most substantial viscous losses. With a *tan(δ)* of 0.6, these are non-negligible. This outcome arises due to the formation of dangling chains within the network when it becomes more off-stoichiometric (Figure 3). Consequently, materials with a lower *r*-value have a poorer dynamic behavior, which can be crucial in different applications. 

When observing the results of dynamic rheometry (Figure 13d–f), it is clear that changing the *r*-ratio largely impacts the (de)gelation temperature (*T_gel_*). A higher r-ratio leads to the narrowing of the degelation transition that is pushed to higher temperatures. Nevertheless, upon looking at the conversion as a function of temperature (Figure 13g), all three polymers follow almost an identical relation. The difference in (de)gelation temperature results from the change in (de)gelation conversion (*x_gel_*) that is affected by the *r*-ratio according to Equation (8). By decreasing the *r*-ratio, *x_gel_* is pushed to higher values. Consequently, polymers with a lower *r*-ratio can be (re)processed at a lower temperature; however, this also means their thermo-mechanical stability is lower. 

In Figure 13h–j, the Diels–Alder (*[DA]_eq_*), furan (*[F]_eq_*), and maleimide (*[M]_eq_*) concentrations are presented as a function of the temperature according to Equation (7). At low and room temperatures, almost all maleimide is reacted, illustrated by an almost zero *[M]_eq_*. However, there is a large number of unreacted furan groups present, shown by *[F]_eq_*, resulting from a low *r*-value. It is this furan content that speeds up the healing through an increase in the reaction rate, as shown by the differential Equation (4). 

#### 3.2.4. Thermal Behaviour as Function of Temperature

**Figure 14:**
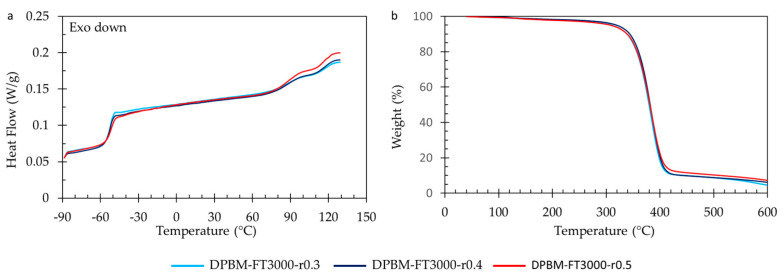
Thermal analysis for the triamine-based Diels–Alder polymers with varying *r*-ratio. (**a**) Differential scanning calorimetry (DSC). (**b**) Thermogravimetric analysis (TGA).

The DSC measurements (Figure 14a) confirm the limited influence of the r-ratio on T_g_. They also indicate that T_g_ only slightly decreases with a decrease in the r-ratio. When examining the high-temperature endothermic peaks (80–130 °C), it is evident that the polymer with the highest r-ratio exhibits the largest endothermic peak. This is attributed to a higher concentration of Diels–Alder bonds that absorb heat upon debonding. Analyzing the TGA results reveals that altering the r-ratio does not affect the degradation profile. However, it can be observed that the more highly crosslinked materials, characterized by a higher r-ratio, display a higher weight content after the initial degradation step, which is attributed to a greater aromatic content present in the bismaleimide.

## 4. Discussion 

Although classified as non-autonomous in the literature [39], Diels–Alder-based reversible polymers exhibit autonomous self-healing of macroscopic damages at ambient conditions, such as room temperature. This paper showed five Diels–Alder networks that could heal within 24 h from significant damage (e.g., being cut in half) and at ambient conditions without the addition of any external heat. However, achieving this requires mobility within the network, which can be attained by reducing the crosslink density. This reduction can be accomplished through two strategies investigated in this paper: increasing the molecular weight of the monomers or decreasing the maleimide-to-furan (*r*) ratio.

In general, reducing the crosslink density, whether through an increase in molecular weight or reduction in the *r*-ratio, results in a faster increase in healing efficiency, e.g., a shorter healing time. However, care should be taken when evaluating the healing performance based on the healing efficiency alone. The healing efficiency is defined as the recovery of the mechanical properties prior to damage, including Young’s modulus, fracture stress, fracture strain, and toughness. The lower these initial mechanical properties are, the faster they can be recovered at room temperature. Materials with a higher crosslink density have a higher Young’s modulus, fracture stress, fracture strain, and toughness; therefore, it takes longer to recover these initial properties after damage. Nevertheless, although the healing efficiency increases lower for these higher crosslinked materials, they can withstand higher stresses at a faster rate. This is attributed to the larger number of Diels–Alder bonds that are formed across the fracture surfaces, resulting from the more densely crosslinked network structure. Depending on the application, one will prefer faster relative (e.g., healing efficiency) or absolute recovery (e.g., fracture stress or toughness) of the mechanical properties. 

The reduction in crosslink density also affects the thermomechanical behavior of the material at higher temperatures. Whether the crosslink density is decreased through adjustments in molecular weight or the r-ratio, the degelation temperature is lowered. Consequently, the material can be reprocessed at lower temperatures, which can be advantageous in terms of energy consumption. However, this decrease in crosslink density also results in reduced temperature resistance, as the material experiences a loss of mechanical and structural stability at lower temperatures. Nevertheless, we present in this paper room temperature healing Diels–Alder polymers that can operate in applications extending up to temperatures of 60 °C or even higher. 

All Diels–Alder networks shown in this paper also exhibit almost instantaneous partial healing minutes after being damaged. The immediate healing occurs due to the presence of reactive maleimide and furan groups at the fracture surfaces, which are formed upon mechanical breaking of the Diels–Alder bonds (cutting). When the fracture surfaces are brought back into contact, these reactive groups exhibit a strong propensity to react. Furthermore, additional adhesive forces and secondary interactions contribute to the initial healing process. The influence of these non-covalent interactions is more pronounced in the elastomer with lower crosslink density. This immediate healing property holds significant value in various future applications as it facilitates the establishment of excellent contact and ensures the cohesion of fracture surfaces throughout the subsequent healing process. In numerous applications, such as self-healing soft robots, as previously published [19], damages are self-sealed through the elastic recovery of the polymeric network (e.g., snap back). Combining this elastic recovery with instantaneous healing enables autonomous self-sealing followed by self-healing, eliminating the need for external or human intervention. Moreover, the rapid autonomous sealing and healing process significantly minimizes the risk of incorporating dirt and dust particles into the healed area, thereby reducing any adverse impact on healing performance.

One major drawback of the presented Diels–Alder polymers, as well as autonomous intrinsic self-healing polymers in general, is that the fracture surfaces must be promptly recombined after damage occurs. Failure to do so results in the separate recombination of the mechanically formed reactive groups on the two fracture surfaces, leading to a loss of autonomous self-healing capability. Nevertheless, if this happens, long gaping damages in the presented Diels–Alder polymers can still be healed using a heat cool cycle, as demonstrated in [40]. This is made possible by the thermal dissociation of Diels–Alder bonds during the heating phase, leading to an increased presence of reactive maleimide and furan groups both within the polymer network and on the fracture surfaces. Upon subsequent cooling, the Diels–Alder bonds reform throughout the network and across the fracture surfaces, ultimately restoring the initial properties. 

The most effective acceleration of healing occurs when the crosslink density is reduced by decreasing the *r*-ratio. Our findings demonstrate that extremely low *r*-values of 0.3 enable Diels–Alder polymers to rapidly heal significant damage and fully restore their initial mechanical performance within a mere five hours at room temperature. The reason for this enhanced healing is an increase in mobility that goes together with an increase in excess of reactive furan. This excess of furan extensively increases the rate of reaction. However, an excessive increase in the amount of furan has adverse effects on other material parameters, which can be crucial depending on the specific application. A higher concentration of unbound furan results in a greater number of dangling chains within the network. These dangling chains have a negative impact on the elastomers’ dynamics, as they contribute to increased viscous portion in their viscoelastic behavior. In applications such as soft robotics, rapid actuation and efficient energy storage are of great importance. The presence of a significant viscous contribution in the viscoelastic behavior introduces time-dependent responses that limit the system’s dynamics, such as actuation speed, and lead to higher energy consumption due to viscous losses. Furthermore, when reducing the crosslink density by adjusting the *r*-value, the spacing between the crosslinks (e.g., the coiled propylene oxide chains) is not increased. As a result, the increase in fracture strain is limited, which in turn, restricts the toughness of the material.

By decreasing the crosslink density through an increase in the molecular weight of the monomers, the healing process is also accelerated, but to a lesser extent. Nonetheless, this approach enables the development of Diels–Alder polymers that can fully restore their performance after significant damage within a single day under normal ambient conditions. However, this method does not compromise other mechanical properties. It does not result in an increased presence of dangling chains, thereby minimizing the occurrence of viscous losses. By utilizing longer monomers, the spacer length between the crosslinks is increased. Consequently, the polymer chains between the crosslinks exhibit more extensive coiling, allowing the material to undergo stretching to longer strains. This augmentation in fracture strain also results in a higher toughness of the material. While reducing the crosslink density through the *r*-ratio has minimal impact on the glass transition temperature (*T_g_*), decreasing it via the molecular weight of the monomers leads to a decrease in *T_g_*. This can also be attributed to the increase in spacer length between the crosslinks.

## 5. Conclusions

In conclusion, caution must be exercised when categorizing intrinsic self-healing polymers into autonomous and non-autonomous (stimulus-dependent) categories, as the ability to self-heal in ambient conditions relies not only on the reversible chemistry but also on the composition of the polymer network. In the case of Diels–Alder polymers, if the primary objective is rapid healing, the preferred approach is to accelerate the process by reducing the crosslink density through a lower maleimide-to-furan ratio (*r*). However, if the material’s mechanical properties, such as fracture strain and toughness, as well as its dynamic performance, are of utmost importance, it is recommended to expedite healing by decreasing the crosslink density through the utilization of monomers with higher molecular weight. Nevertheless, both techniques demonstrate the remarkable effectiveness of fast room temperature self-healing in Diels–Alder polymers, thereby invalidating the previous classification of these materials as non-autonomous.

## Figures and Tables

**Table 1:** Triamine-based and diamine-based Diels–Alder network with different Diels–Alder concentrations at room temperature.

Monomers	*f*	*M/f* (g.mol^−1^)	Elastomers	*r*	*[M]*_0_ (mol.kg^−1^)	*[F]*_0_ (mol.kg^−1^)	*[DA]_eq,_*_25 °C_ (mol.kg^−1^)	*[M]_eq,_*_25 °C_ (mol.kg^−1^)	*[F]_eq,_*_25 °C_ (mol.kg^−1^)
DPBM	2	179							
FT5000	6	1112	DPBM-FT5000	0.5	0.41	0.83	0.61	0.01	0.42
FT3000	6	691	DPBM-FT3000	0.5	0.63	1.27	0.63	0.01	0.64
FD4000	4	1297	DPBM-FD4000	0.6	0.43	0.71	0.43	0.01	0.28
FD2000	4	609	DPBM-FD2000	0.6	0.83	1.38	0.83	0.01	0.55

**Table 2:** Triamine-based Diels–Alder network with different maleimide-to-furan ratio *r*.

Monomers	*f*	*M/f* (g.mol^−1^)	Elastomers	*r*	*[M]*_0_ (mol.kg^−1^)	*[F]_*0*_* (mol.kg^−1^)	*[DA]_eq,_*_25 °C_ (mol.kg^−1^)	*[M]_eq,_*_25 °C_ (mol.kg^−1^)	*[F]_eq,_*_25 °C_ (mol.kg^−1^)
DPBM	2	179							
FT3000	6	691	DPBM-FT3000	0.3	0.40	1.34	0.40	0.01	0.94
FT3000	6	691	DPBM-FT3000	0.4	0.52	1.30	0.52	0.01	0.78
FT3000	6	691	DPBM-FT3000	0.5	0.64	1.27	0.64	0.01	0.63

## Supplementary Materials

The following supporting information can be downloaded at: https://www.mdpi.com/article/10.3390/polym15173527/s1, Figure S1: NMR of Jeffamine D2000. The calculated molar mass of D2000 is 1792 g.mol^−1^; Figure S2: NMR of Jeffamine D4000. The calculated molar mass of D4000 is 4546 g.mol^−1^; Figure S3: NMR of Jeffamine T3000. The calculated molar mass of T3000 is 3180 g.mol^−1;^ Figure S4: NMR Jeffamine T5000-1. The calculated molar mass of T5000-1 is 5709 g.mol^−1^. Supporting video on the fast room temperature healing can be found at https://www.youtube.com/watch?v=a-bCetuPKjs (accessible since 20 July 2023).
