# Supplementary material for: Fast Self-Healing at Room Temperature in Diels–Alder Elastomers
Source: Polymers (Basel). 2023 Aug 24;15(17):3527. doi: 10.3390/polym15173527 (PMC10490179; doi:10.3390/polym15173527)
Supplement: Supplementary file 1 [file polymers-15-03527-s001.zip › polymers-2543000-supplementary.pdf]

polymers MDPI

Supporting Information: NMR Spectra on Jeffamines

The molar masses of the Jeffamines were determined by nuclear magnetic resonance. The spectra, including the calculations of the molar masses, can be found in the figures below.

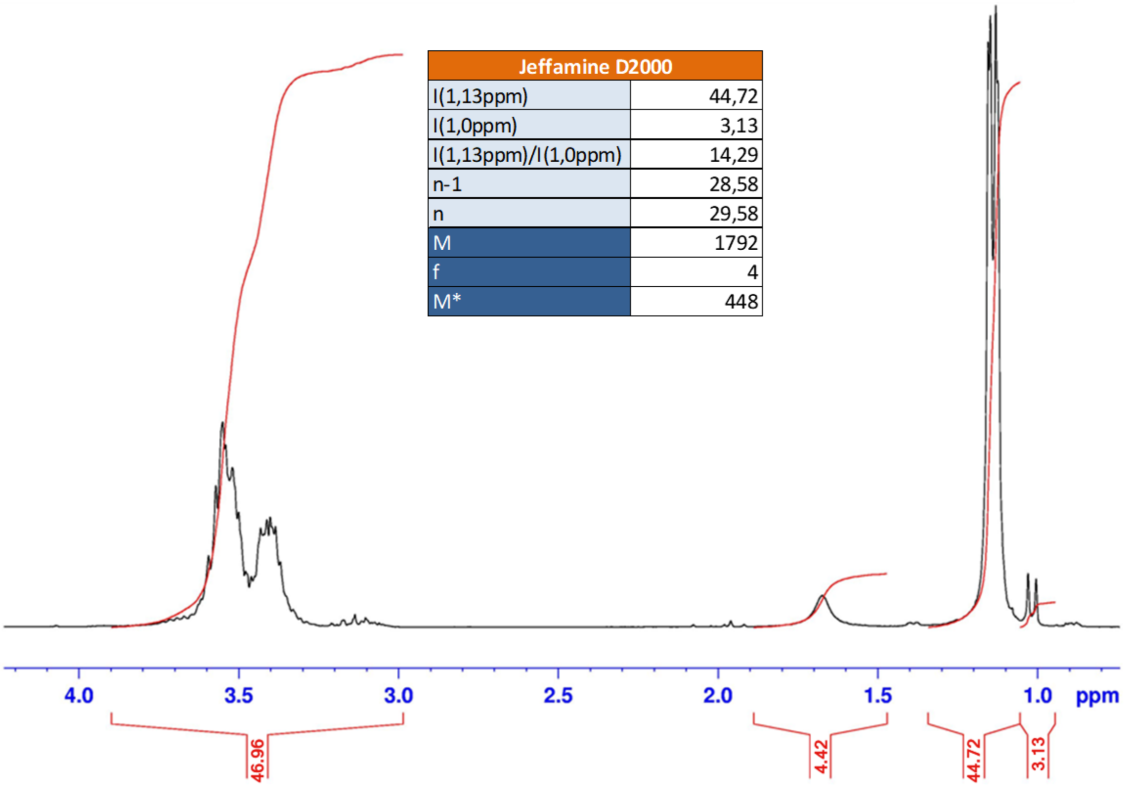

Jeffamine D2000	
I(1,13ppm)	44,72
I(1,0ppm)	3,13
I(1,13ppm)/I(1,0ppm)	14,29
n-1	28,58
n	29,58
M	1792
f	4
M*	448

Figure S1. NMR of Jeffamine D2000. The calculated molar mass of D2000 is 1792 g.mol^{-1}.

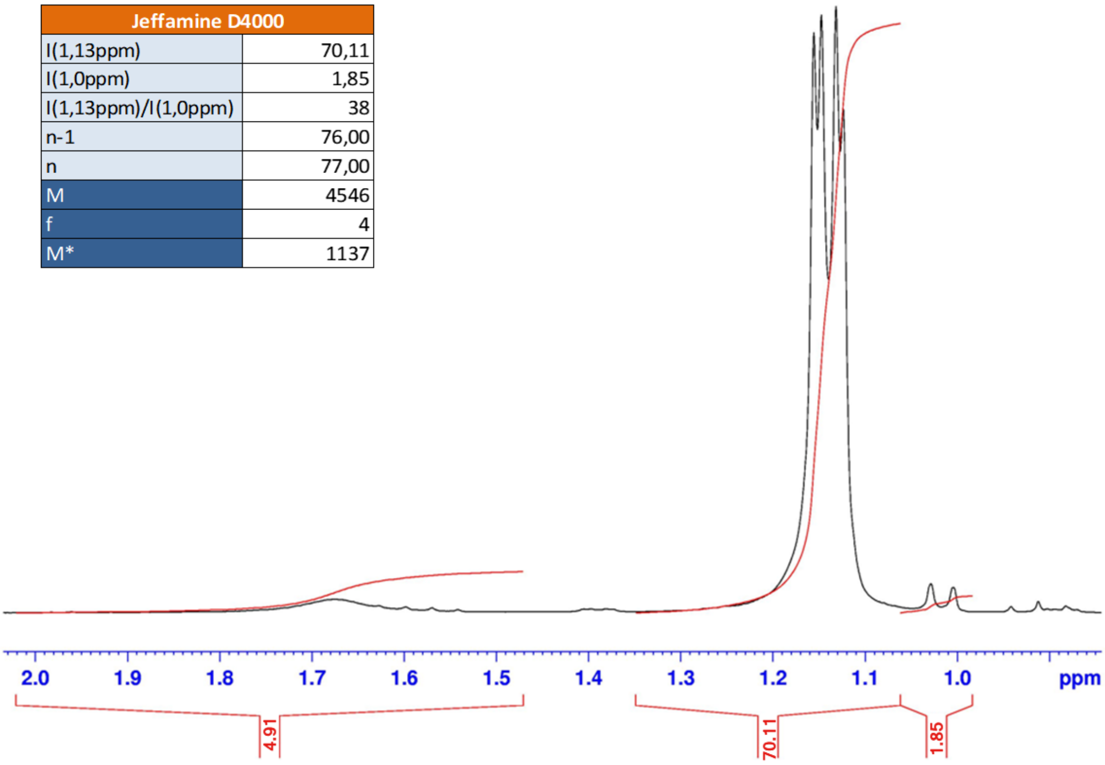

Jeffamine D4000	
I(1,13ppm)	70,11
I(1,0ppm)	1,85
I(1,13ppm)/I(1,0ppm)	38
n-1	76,00
n	77,00
M	4546
f	4
M*	1137

µ

Figure S2. NMR of Jeffamine D4000. The calculated molar mass of D4000 is 4546 g.mol^{-1}.

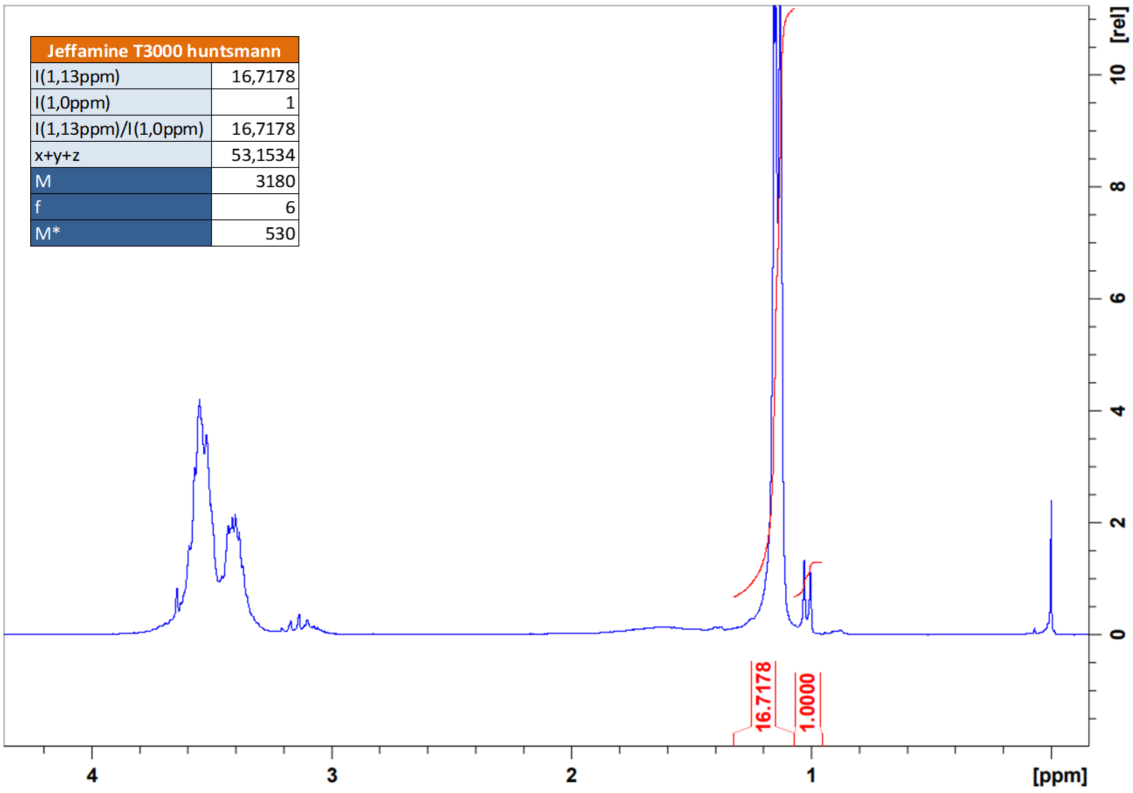

Jeffamine T3000 huntsmann	
I(1,13ppm)	16,7178
I(1,0ppm)	1
I(1,13ppm)/I(1,0ppm)	16,7178
x+y+z	53,1534
M	3180
f	6
M*	530

Figure S3. NMR of Jeffamine T3000. The calculated molar mass of T3000 is 3180 g.mol^{-1}.

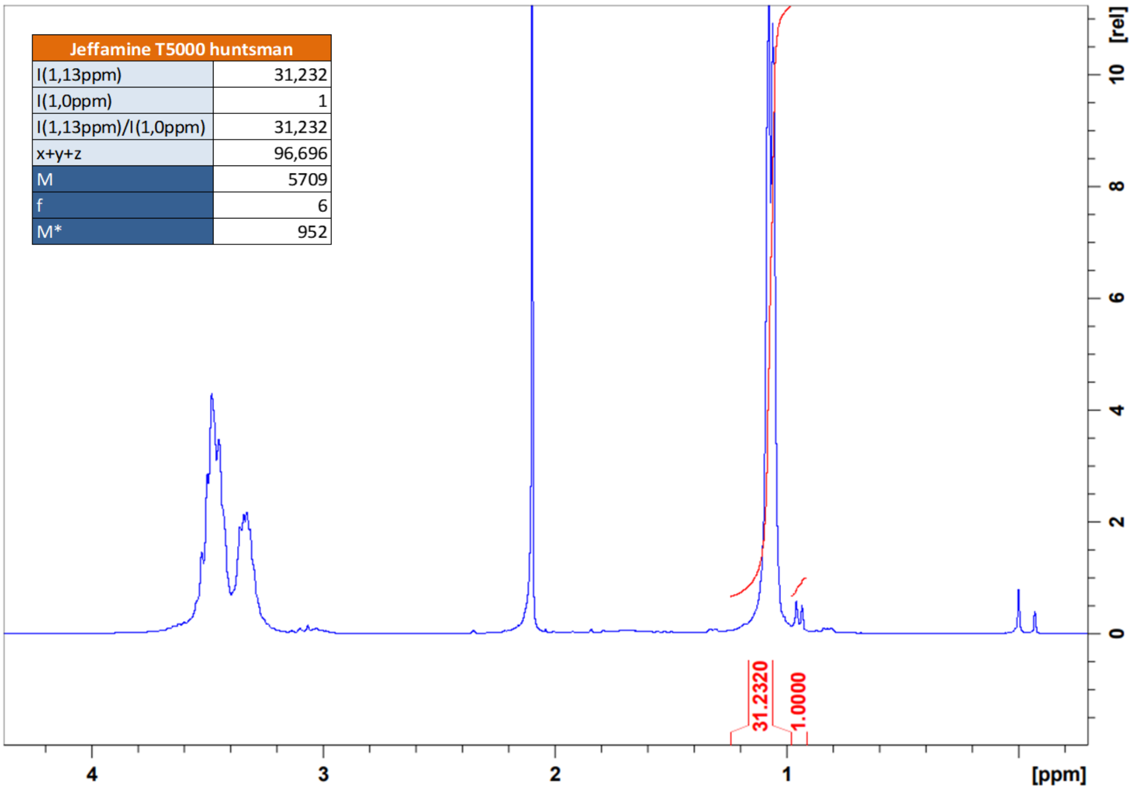

Figure S4. NMR Jeffamine T5000-1. The calculated molar mass of T5000-1 is 5709 g.mol^{-1}.
